# World Bank and the Global Financing Facility

**DOI:** 10.1136/bmj.j3395

**Published:** 2017-08-31

**Authors:** Genevie Fernandes, Devi Sridhar

**Affiliations:** 1 Medical School, Edinburgh University, Edinburgh, UK

## Abstract

In the fourth article of the series, **Genevie Fernandes** and **Devi Sridhar** describe the bank’s new investment model for advancing reproductive, maternal, newborn, child, and adolescent health and nutrition

At the World Economic Forum this year, World Bank President Jim Kim proposed the Global Financing Facility (GFF) to donors as an innovative model for investing in reproductive, maternal, newborn, child, and adolescent health and nutrition (RMNCAH-N).^1^ The World Bank believes that business as usual is not enough to close the annual financing gap of $33.3bn (£25.4bn; €28.4bn) to meet the 2030 sustainable development goals for RMNCAH-N.^2^ Its latest offering—the GFF—is designed as a catalyst to close this gap, as every dollar invested by donors will be linked with $4 of bank credits, multiplying the effect of donor contributions in countries where action is needed the most.^2^ Since its inception in July 2015 and implementation in seven high burden countries to date,^3^ ^4^ the GFF has been lauded and criticised in equal measure.^5^ ^6^ In this article, we explain the origins and mechanism of the GFF, and discuss the benefits and some initial concerns about this investment model.

## Origins of the GFF

The GFF is a multidonor trust fund managed by the World Bank with financial commitments from bilateral donors and private foundations of more than $1bn (fig 1).^7^ The GFF is based on the existing Health Results Innovation Trust Fund (HRITF) managed by the World Bank and supported by Norway and the UK through commitments of $575m from 2007 to 2022.^8^

**Figure f1:**
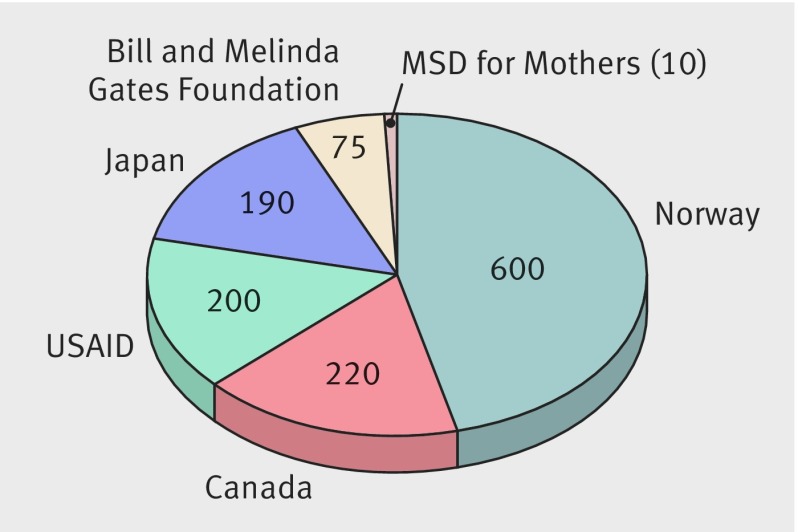
**Fig 1** Main contributions to the Global Financing Facility in $m^8^ (MSD=Merck for Mothers)

The HRITF supports results based financing interventions whereby providers are paid on achieving planned indicators to improve the coverage and quality of maternal and child health services. Country programmes under the HRITF are financed by linking grants from the trust fund with credit from the World Bank’s concessional lending arm—the International Development Association (IDA).^8^ Evaluation of the HRITF showed that while results based financing improves service coverage and quality, albeit with variations across interventions, the key recommendation of a strategic, scaled, and sustainable framework that views results based financing as an entry point for tackling health system problems is not always easy to implement, especially in weak health systems.^8^ ^9^ ^10^ The GFF grew out of this recommendation under the leadership of World Bank president Jim Kim and Tim Evans, the senior director of the health, nutrition, and population sector.^11^ ^12^

## Mechanism and governance of the GFF

The GFF retains two key features of its precursor—the HRITF. Firstly, the model focuses on results, and, secondly, it links grants with credits from the World Bank’s lending arms—the IDA and the International Bank for Reconstruction and Development (IBRD).

Globally, the GFF seeks finance from donors to be disbursed as grants, and nationally, it links these grants with credits from the IDA or IBRD for RMNCAH-N projects in 62 high burden, low, and lower middle income countries.^2^ For each $1 of grant, the GFF matches around $4 in credits from the IDA or IBRD, depending on the income level of the recipient country. This translates to a financial arrangement whereby countries choosing to invest credits from their national IDA/IBRD allocation in RMNCAH-N projects will be offered a grant from the GFF trust fund. While the grant encourages countries to use their IDA/IBRD credits for RMNCAH-N, this spending is substitutive and does not provide additional public expenditure in this area, as IDA/IBRD credits are essentially a country’s own resources, although borrowed, which are invested in RMNCAH-N instead of other sectors. However, the GFF aims to form country driven partnerships for aligning financial resources from the GFF with additional investments from government, development, and private partners to meet RMNCAH-N goals.^2^

The governance of the GFF gives substantial decision making authority to the bank and the donors. At the heart of this structure is an investors group, which mobilises financing, and within this group is the trust fund committee, that decides which countries and projects are funded (fig 2). A GFF secretariat, staffed within the bank, manages and monitors the trust fund. GFF trust fund financing is integrated into IDA/IBRD country projects approved by the World Bank board.^13^

**Figure f2:**
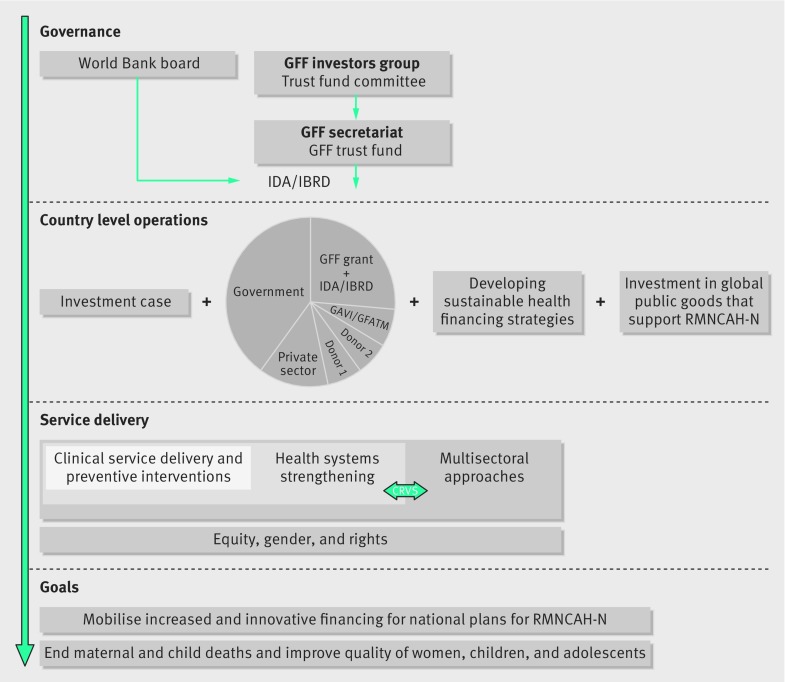
**Fig 2** Framework of the Global Financing Facility, adapted from the Global Financing Facility (GFF)=business plan.^3^ CRVS= civil registration and vital statistics; GFATM= Global Fund to Fight AIDS, Tuberculosis and Malaria; IBRD=International Bank for Reconstruction and Development; IDA=International Development Association; RMNCAH-N=reproductive, maternal, newborn, child, and adolescent health and nutrition.

Membership of the investors group is based on financial or in-kind (technical or advocacy based) contributions, and institutional authority to align resources for RMNCAH-N projects, while donors form the trust fund committee members.^13^ The investors group is chaired by the president of the global development programme of the Bill and Melinda Gates Foundation, and comprises one or two representatives from recipient and donor governments, international organisations (Gavi (the Vaccine Alliance) and the Global Fund to Fight AIDS, Tuberculosis and Malaria), private organisations (Merck for Mothers, Grand Challenges Canada, and Philips), private foundation (Gates), civil society (African Health Budget Network, Plan International, Population Council, RESULTS, and World Vision), and multilateral organisations (Unicef, UNFPA, World Bank, and WHO).^14^

## Mobilising money for the GFF

Sixty two high burden countries that are willing to invest their IDA/IBRD funds in RMNCAH-N projects can apply for a GFF package. An investment case is the starting point of the GFF process. World Bank country staff work with recipient governments to develop an investment case, which identifies areas for action, corresponding obstacles, appropriate evidence based interventions, and costing, with an emphasis on alignment with national priorities. Design of the investment cases is financed by the GFF trust fund. The GFF trust fund committee and the World Bank board review the case and decide on approval and disbursement of funds.^2^ As of April 2017, 16 countries had begun the GFF process and nine country projects have been approved, with a total commitment of $292m in grants and $1301m in IDA/IBRD financing^4^ ^15^ (table 1). While 12 of the 16 GFF countries received funds from the HRITF, the criteria for selecting frontrunner countries for GFF financing are unclear.

**Table 1 tbl1:** Approved financial commitments from the GFF trust fund and IDA/IBRD 16

Recipient country	GFF Trust Fund $m	IDA/IBRD $m
Cameroon	27	100
Democratic Republic of Congo	50	350
Ethiopia	60	150
Guatemala	9	100
Kenya	40	150
Liberia	16	16*
Nigeria	20	125
Tanzania	40	200
Uganda	30	110
**Total**	**292**	**1301**

*To be confirmed. GFF=Global Financing Facility; IBRD=International Bank for Reconstruction and Development; IDA= International Development Association.

The GFF mobilises finances in four ways. Firstly, complementary financing is employed, whereby partners (donors) with in-country programmes, such as GAVI and the Global Fund, are encouraged to align their financial resources to meet mutual RMNCAH-N goals, thereby increasing efficiency and avoiding duplication of efforts. Secondly, the GFF works to increase government expenditure on RMNCAH-N through mechanisms ranging from technical assistance in managing public finances to making mobilisation of domestic resources a legal requirement. Thirdly, GFF grants are matched with credits from IDA/IBRD. The fourth route enlists domestic and international private sector resources through pathways such as development impact bonds, whereby investors provide capital for an intervention to reach planned outcomes, and funders (government and donors) pay only when the intervention succeeds.^2^

## Interventions covered by the GFF

The GFF finances preventive and clinical interventions for RMNCAH-N, health systems strengthening, and multisectoral projects, with demonstrated effectiveness and focus on dealing with equity, gender, and rights. Apart from mobilising financing for the investment case, the GFF also works with countries rising from low to middle income status and thereby graduating from IDA to IBRD, to develop sustainable health financing plans. The GFF is building a global evidence base for health financing strategies for RMNCAH-N, and a centre of excellence on civil registration and vital statistics using funding from the Canadian government.^2^ The GFF will invest in strengthening national monitoring and evaluation systems. It will include independent evaluations at the national and global level measuring the short term impact on efficiency, domestic resource mobilisation, and donor alignment, and the long term effect on coverage of interventions and health outcomes.^4^

## Advantages of the GFF model

The GFF is 23 months old and still a work in progress. Nevertheless, there are five reasons why it could become a game changer in financing for maternal, child, and adolescent health and nutrition. Firstly, the GFF has the support of political leaders from leading donor and recipient countries and from the heads of key donor organisations, including the Gates Foundation.^5^ Secondly**,** this model uses RMNCAH-N as an entry point for ensuring a basic healthcare package for women, children, and adolescents through a strengthened primary healthcare delivery system, thereby accelerating country level efforts towards universal health coverage.^15^ Thirdly**,** it invests in broader health systems strengthening, such as the health workforce, supply chain management, and information systems, while also including multisectoral investments in education, water supply, and sanitation, which aid the upstream determinants of health and lead to improvements in population health.^2^ Fourthly**,** by specifically including adolescents, who have previously been overlooked, the GFF can tackle preventable and treatable sexual and reproductive health problems, resulting in health gains for this group in later years. Finally**,** the GFF can use the bank’s and financial expertise, coupled with political backing, to support governments in domestic resource mobilisation for RMNCAH-N.

## Concerns about the GFF model

This investment model is not without potential disadvantages. Having the traditional set of donor agencies making key decisions can influence the selection of countries, choice of interventions, and disbursement of funds. Although this limitation has been tackled to an extent by the recent approval of the civil society engagement strategy,^16^ ^17^ a detailed action plan needs to be rolled out across all national GFF projects to ensure stronger civil society involvement. 

Although the GFF’s attempt to bring all national stakeholders and donors around the table advances the agenda of aligning goals and harmonising financial resources for RMNCAH-N, it may also become a risk to implementation. For instance, donors within a country may not be willing to commit to complementary financing based on the investment case, and development of a strong investment case itself is contingent on the capacity of the bank staff and the recipient government counterparts and the inter-relationships between the two. Mitigation of such risks needs to be built into the GFF.

The GFF focuses on results, and in investment cases of some countries, such as Ethiopia, it links disbursement with the achievement of progress indicators.^15^ This can be problematic if measures are not built in to overcome any negative effects of failure to achieve results, ranging from demotivation of health workers to irregular payments. Furthermore, although grants have stimulated potential domestic resources in some cases, there is a risk that increases in external assistance might displace domestic government health spending.^18^ The GFF can mitigate this risk by monitoring government health expenditures and establishing collaborative (and not prescriptive) goals based on the country context, to maintain or increase public spending.

If the GFF does attract increased contributions from sovereign bilateral donors, this shift in financing could also affect core contributions to the IDA and IBRD replenishments and, subsequently, project funding for other health areas. Furthermore, while leveraging and multiplying the effect of their contributions may be valuable for bilateral donors, foundations, and philanthropic groups, involvement from the private sector will require return on investment, and this is an area which the GFF will need to explore and fine tune its approach based on lessons from the frontrunner countries.

## Conclusion

The World Bank’s involvement in maternal and child health has evolved from family planning in the 1970s^19^ to child survival and safe motherhood in the 1980s,^20^ to advocating reproductive and child health in the 1990s,^21^ to more recently, adopting the RMNCAH approach covering life course interventions for women, children, and adolescents.^2^ With the addition of an ‘N’ to include nutrition, it is increasingly clear that the comprehensive RMNCAH-N framing could be the bank’s strategy to broaden the appeal of investments in strengthening health systems. The GFF presents an attractive avenue for such investments, with an emphasis on domestic resources. This investment model also takes the bank into the heart of domestic resource mobilisation by allowing it to work closely with governments on improving efficiency and revenue generation, and prioritising health in budgets.

**Key messages**

The Global Financing Facility (GFF), a multidonor trust fund, is the World Bank’s latest investment model aimed at closing the annual financing gap of $33.3bn to meet the 2030 sustainable development goals for reproductive, maternal, newborn, child, and adolescent health and nutrition (RMNCAH-N)
The GFF offers 62 high burden countries grants if they agree to invest their IDA or IBRD credits in results focused RMNCAH-N interventions, thereby matching each $1 of grant with $4 of bank finance
Benefits of the GFF include promotion of universal health coverage and strengthening of health systems through increased mobilisation and harmonisation of development financing and domestic public and private resources.
While the GFF model incentivises borrowing for RMNCAH-N, it also works with countries rising from low to middle income status to develop sustainable strategies for increasing domestic financing

## Notes

Contributors and sources: GF is a researcher from India, and currently, a PhD student at the University of Edinburgh, studying the role and influence of the World Bank in maternal and child health over the past four decades. DS holds a Wellcome Trust investigator award on the role of the World Bank in global health and is the coauthor of *Governing Global Health: Who Runs the World and Why? *(OUP, 2017). Data analysed for this series included World Bank financial datasets, archival sources, publications and reports, and staff interviews. GF collected the data, analysed it, and drafted the initial version of the paper. DS helped conceptualise and design the study and revised the draft.

Provenance and peer review: Commissioned; externally peer reviewed.
